# Concerns regarding NGAL as a biomarker for heart failure without kidney disease

**DOI:** 10.34172/jcvtr.026.33669

**Published:** 2026-03-30

**Authors:** Çağrı Zorlu, Sefa Erdi Ömür

**Affiliations:** Department of Cardiology, Tokat Gaziosmanpasa University Hospital, Tokat, Turkey

## Dear Editor,

 We appreciate the study by Asadolahi Mashhadian et al exploring NGAL as a biomarker for early heart failure (HF) diagnosis in patients without kidney disease.^1^ However, several concerns regarding inconsistencies, literature discrepancies, and methodological limitations warrant clarification.

 The study claims patients had no kidney disease, with glomerular filtration rate (GFR) > 59 mL/min, yet reports significantly elevated creatinine (1.22 mg/dL vs. 0.23 mg/dL, *P* < 0.001) and blood urea nitrogen (BUN) (23.21 mg/dL vs. 14.14 mg/dL, *P* < 0.001) in the HF group. These levels approach renal impairment thresholds (e.g., creatinine > 1.5 mg/dL, per citation 3), raising questions about patient selection criteria. How were “no kidney disease” patients defined, given these renal parameter elevations? Additionally, NGAL’s predictive value for HF is significant in logistic regression (*P* = 0.002), but no correlation exists with cardiac troponin I (CTNI) or C-reactive protein (CRP), suggesting NGAL may reflect non-cardiac pathways, possibly renal stress.

 The study’s findings contrast with literature indicating NGAL’s elevations in HF are often linked to renal dysfunction or inflammation.^2,3^ Shrestha et al suggest NGAL is primarily driven by renal stress in HF^3^, which aligns with the elevated creatinine and BUN observed. Could the authors clarify how NGAL’s role in HF was distinguished from renal involvement?

 Methodologically, the cross-sectional design limits claims of “early diagnosis,” as patients were already diagnosed with HF with reduced ejection fraction (EF < 40%). A prospective study would better support early detection claims. The assumption of data normality lacks justification, and the logistic regression model’s wide confidence interval for creatinine (0.302–1426.701) suggests instability. Can the authors provide model diagnostics? The small sample size (n = 118) lacks a power calculation, and blood sampling timing relative to HF onset is unclear, potentially affecting NGAL levels.^4^

 We commend the authors’ effort but seek clarification on patient selection, NGAL’s specificity for HF, study design, and statistical robustness to strengthen the findings.

## Competing Interests

 The author declares no conflict of interest in this study.

## Ethical Approval

 Not applicable.
